# A pyramidal deep learning pipeline for kidney whole-slide histology images classification

**DOI:** 10.1038/s41598-021-99735-6

**Published:** 2021-10-12

**Authors:** Hisham Abdeltawab, Fahmi Khalifa, Mohammed Ghazal, Liang Cheng, Dibson Gondim, Ayman El-Baz

**Affiliations:** 1 Bioengineering Department, University of Louisville, Louisville, KY USA; 2 Department of Electrical and Computer Engineering, Abu Dhabi University, Abu Dhabi, United Arab Emirates; 3 Department of Pathology, Indiana University School of Medicine, Indianapolis, IN USA; 4 Department of Urology, Indiana University School of Medicine, Indianapolis, IN USA; 5 Department of Pathology and Laboratory Medicine, University of Louisville, Louisville, KY USA

**Keywords:** Cancer, Diseases, Oncology, Engineering

## Abstract

Renal cell carcinoma is the most common type of kidney cancer. There are several subtypes of renal cell carcinoma with distinct clinicopathologic features. Among the subtypes, clear cell renal cell carcinoma is the most common and tends to portend poor prognosis. In contrast, clear cell papillary renal cell carcinoma has an excellent prognosis. These two subtypes are primarily classified based on the histopathologic features. However, a subset of cases can a have a significant degree of histopathologic overlap. In cases with ambiguous histologic features, the correct diagnosis is dependent on the pathologist’s experience and usage of immunohistochemistry. We propose a new method to address this diagnostic task based on a deep learning pipeline for automated classification. The model can detect tumor and non-tumoral portions of kidney and classify the tumor as either clear cell renal cell carcinoma or clear cell papillary renal cell carcinoma. Our framework consists of three convolutional neural networks and the whole slide images of kidney which were divided into patches of three different sizes for input into the networks. Our approach can provide patchwise and pixelwise classification. The kidney histology images consist of 64 whole slide images. Our framework results in an image map that classifies the slide image on the pixel-level. Furthermore, we applied generalized Gauss-Markov random field smoothing to maintain consistency in the map. Our approach classified the four classes accurately and surpassed other state-of-the-art methods, such as ResNet (pixel accuracy: 0.89 Resnet18, 0.92 proposed). We conclude that deep learning has the potential to augment the pathologist’s capabilities by providing automated classification for histopathological images.

## Introduction

Kidney cancer is a major health problem due to morbidity, mortality, and health care costs. In 2021, the American Cancer Society estimates the occurrence of 76,080 new cases of kidney cancer and 13,780 deaths^1^. Renal cell carcinoma (RCC) is the most common type of kidney cancer, and is a heterogeneous group of malignancies originating from epithelial cells of the kidney parenchyma. There are more than 10 subtypes of RCC^2^. These subtypes have distinct molecular characteristics, responses to treatment, and clinical outcomes^3^. The most prevalent subtypes of RCC are clear cell (70–80%), papillary (14–17%), chromophobe (4–8%), and clear cell papillary (4%)^4^. Classification of RCC subtypes is primarily based on morphological features observed on histopathological hematoxylin and eosin–stained slides. Among the four subtypes, the possibility of morphologic overlap is greatest for a subset of clear cell RCC and clear cell papillary RCC cases. This is because both types of tumors can be exclusively comprised of clear cells and can have a predominant tubular/alveolar architecture. Some difficult cases can be accurately diagnosed by an experienced pathologist based on confident identification of distinctive histologic features. Immunohistochemistry can also facilitate the diagnosis since the two tumor types tumors typically have a distinct immunoprofile. Distinction between these two subtypes of RCC is crucial because of prognostic and treatment implications. Clear cell RCC tends to portend poor prognosis with high potential of becoming metastatic^2^. In contrast, clear cell papillary RCC is an indolent tumor with minimal risk of recurrence or metastasis^2^. There is only one case of metastatic clear cell papillary RCC in the literature^5^. Experts in kidney tumor pathology are limited and immunohistochemistry may not be readily available in a low resource setting. Therefore, there is a need to develop techniques that would facilitate this diagnostic process. Pathology is transitioning to become a digital discipline and artificial intelligence–based image analysis techniques are showing promising results to augment pathologist’s capabilities^6^. Deep learning for RCC classification has been reported previously^7^, but the authors only included the three most common types of RCC which have little morphologic overlap. We propose a pyramidal deep learning pipeline for kidney whole slide image (WSI) histology classification to address this challenging task, using a computer-aided diagnostic system of convolutional neural networks (CNNs) for the automated classification of kidney tissues (data and non-tumoral kidney tissues), to separate clear cell RCC from clear cell papillary RCC.

Our framework has the following contributions:

- The first study to discriminate between clear cell RCC and clear cell papillary RCC. This classification has high clinical relevance.
- We propose a pyramidal deep learning model that utilizes a hierarchy of three CNNs that process different image sizes. The deep learning improves the precision of the diagnosis and decreases human error. Furthermore, it delivers reproducible results and an objective assessment.
- Our approach can provide both patchwise and pixelwise classifications.
- We incorporated a statistical approach based on generalized Gauss-Markov random field (GGMRF) to remove inconsistencies in the final pixelwise classification.

## Materials and methods

**Figure 1:**
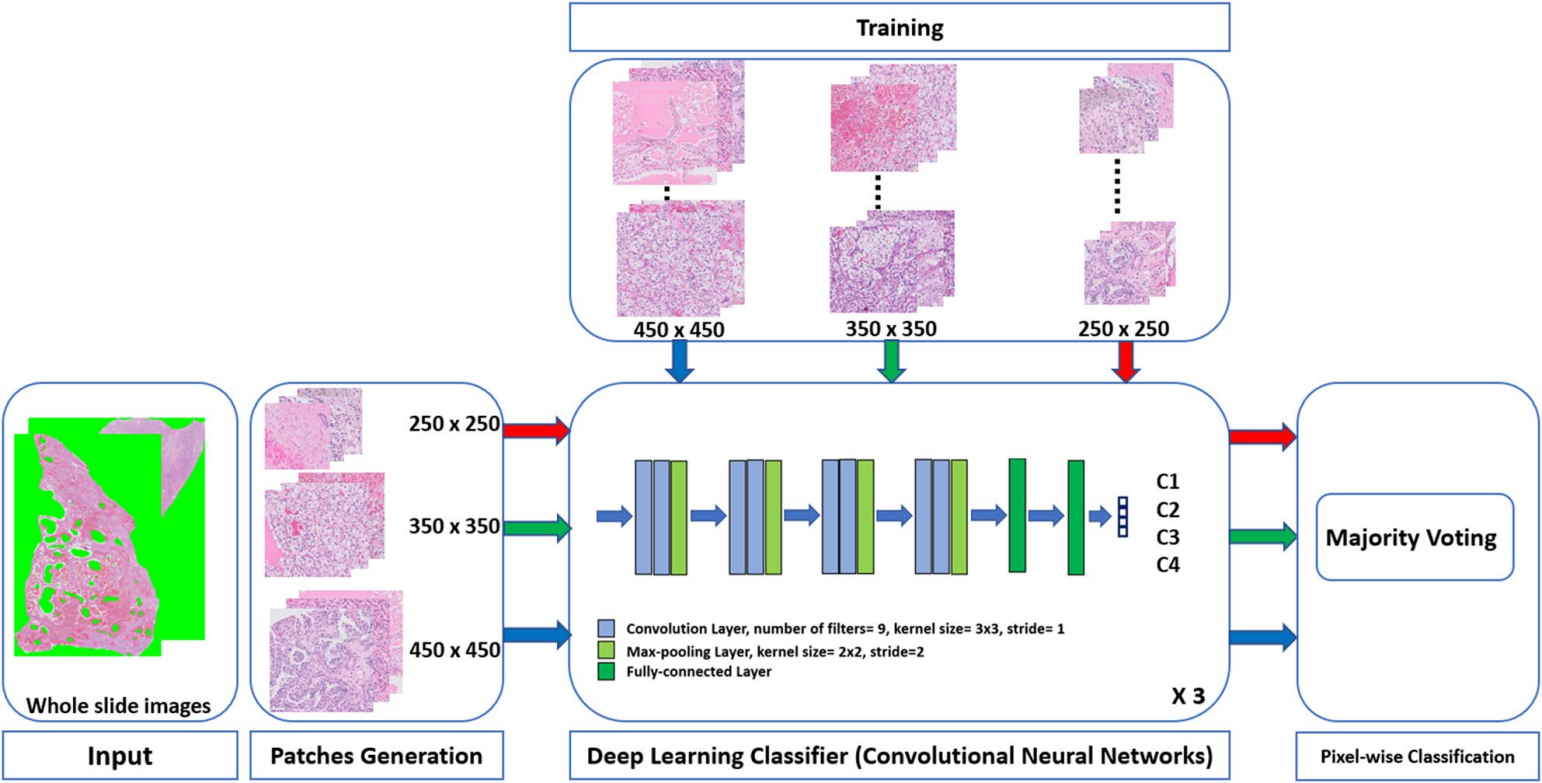
Schematic illustration of the proposed computer aided diagnostic system for automated classification of kidney tissues.

We proposed a computer-aided diagnostic system (Fig. 1) based on deep learning for the automated classification of four kidney tissues: fat (Class 1), parenchyma (Class 2), clear cell papillary RCC (Class 3), and clear cell RCC (Class 4). We started with dividing the WSI into small patches to be fed to the CNNs. These patches were preprocessed to enhance their visual appearance and features, and then used to train and test our deep learning framework.

This study used de-identified digital images (whole slide images) acquired from archived pathology specimens at Indiana University. The dataset consists of images and associated histological findings only. Collection and analysis of this data was approved by the Institutional Review Boards (IRB) of Indiana University, USA (IRB 1301010350) and of University of Louisville, USA (IRB 19.0778). This study was exempt from informed consent by both IRBs. Before digitization, we de-identified all of the hematoxylin and eosin–stained slides. We removed all patient identifying links from the used image files. From the institution files, we randomly chose 30 cases diagnosed with clear cell RCC and 22 cases diagnosed with clear cell papillary RCC. A pathologist with expertise in genitourinary pathology reviewed these cases. We selected one representative slide from each case. Then, we selected 7 slides for the parenchyma tissue type and 5 slides for the fat tissue type. We scanned the slides with a Philips UFS. A pathologist manually segmented the images into four tissue classes: fat, renal parenchyma, clear cell RCC, and clear cell papillary RCC.

### Patch generation

**Figure 2:**
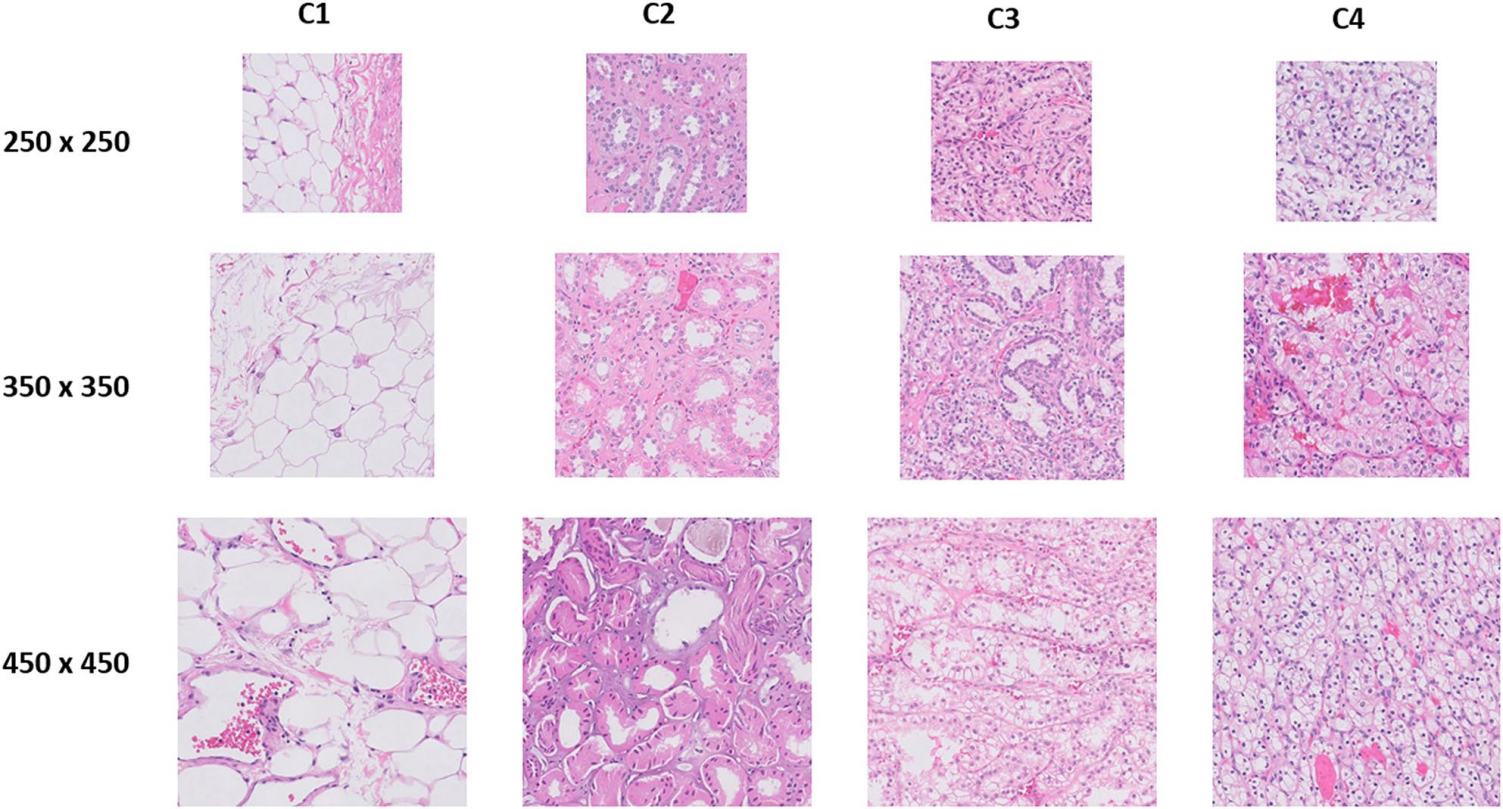
Samples of the generated patches from different image slides at different patch sizes. Note that C1, C2, C3, and C4 refer to Class 1, Class 2, Class 3, and Class 4, respectively.

Our dataset consisted of 64 whole slide images (30 clear cell RCC, 22 clear cell papillary RCC, 7 parenchyma, and 5 fat). To follow the best practice in validating our deep learning framework, the data were divided into two sets (Supplemental Table S1): Set 1 for training and testing (44 WSI) and Set 2 for final validation (20 WSI). The image slides are too large to be processed by a CNN. Therefore, generating image patches to form the image slides produces a suitable image size for the CNN and creates the high sample size necessary for obtaining accuracy from the deep learning framework. Overlapping patches were generated from Set 1 WSI using the three patch sizes: small = 250 × 250, medium = 350 × 350, and large = 450 × 450. A folder for every image slide was created, each containing folders for the three specific patch sizes. A 50% overlap was kept between each patch and the next one. The overlap between patches resulted in learning multiple viewpoints within the tissue by the deep learning framework. Patches that were dominated by background pixels were removed, resulting in approximately 70% available patches for training and the reminder for testing. Figure 2 shows samples for the patches of the Set 1 (training) at different sizes. Overlapping patches were generated for Set 2 (validation). Again, for each image slide, the three different sizes were stored in individual folders. The degree of overlap was higher than of the first set; there was a five-pixels shift in both dimensions between each patch. During inference by our deep learning framework, we assign a label for each input patch and obtained multiple labels for the same pixel because a pixel can belong to multiple patches.

### Preprocessing

To enhance the visual appearance of the patches, we applied two preprocessing techniques for the two datasets: adaptive histogram equalization, followed by edge enhancement.

#### Adaptive histogram equalization

**Figure 3:**
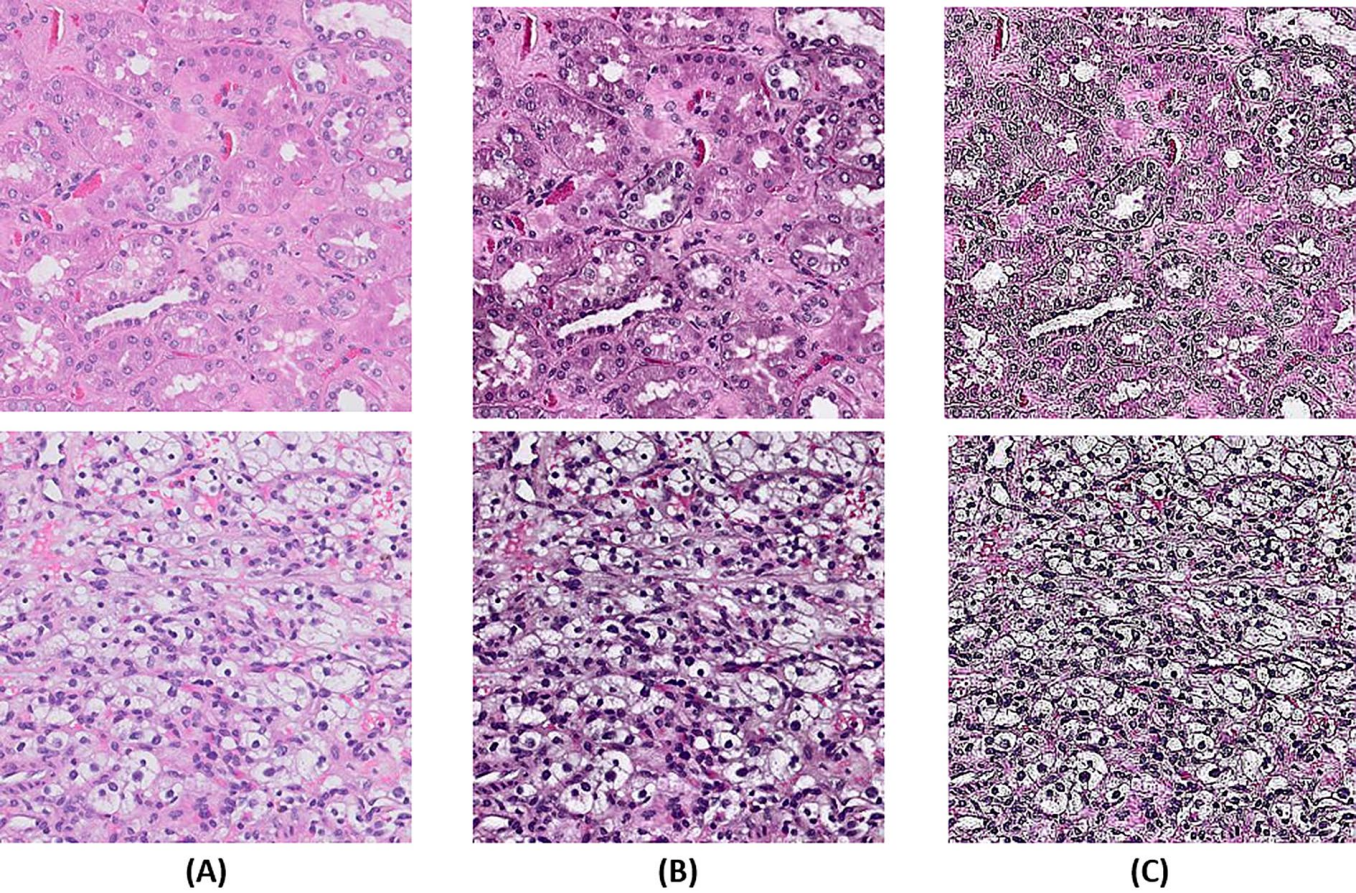
Examples of preprocessing step where original patches are shown in (**A**) and their histogram equalized versions are in (**B**) followed by edge enhancement (**C**).

Adaptive histogram equalization, an image processing technique, was used to improve the contrast of the image patches^8^. A standard histogram equalization generates only one histogram for the whole image; however, adaptive histogram equalization generates multiple histograms, from different parts in the image, which are used to redistribute the pixel values of the image. By doing this redistribution, the local contrast and the edges are enhanced in each part of the image. However, adaptive histogram equalization is prone to noise amplification within homogeneous regions of the image where the histogram is highly concentrated. To prevent noise amplification, a variant was utilized that limits the amplification. This variant is called contrast limited adaptive histogram equalization. After the equalization step in contrast limited adaptive histogram equalization, the artificially generated boundaries were removed by employing bilinear interpolation between neighboring regions (Fig. 3A,B).

#### Image edge enhancement

The edges of important objects were enhanced in image patches from the two datasets (Fig. 3A,C). Resulting in improvement of the visual perception of the image patches from the suppression of low frequency components. This high-pass filtering can be done in the spatial or the frequency domain; we filter the image patches by performing a convolution with a sharpening filter (Supplemental Figure S1) in the spatial domain. For example, if filter is *h*(*m,n*) and the input image patch is *x*(*m,n*), then the filtered image patch is given by Eq. (1).

1 $$ y(m,n) = h(m,n) * x(m,n) $$

where ∗ is the convolution operator. Figure 3 shows examples for edge enhancement of the image patches.

### The proposed deep learning framework

**Figure 4:**
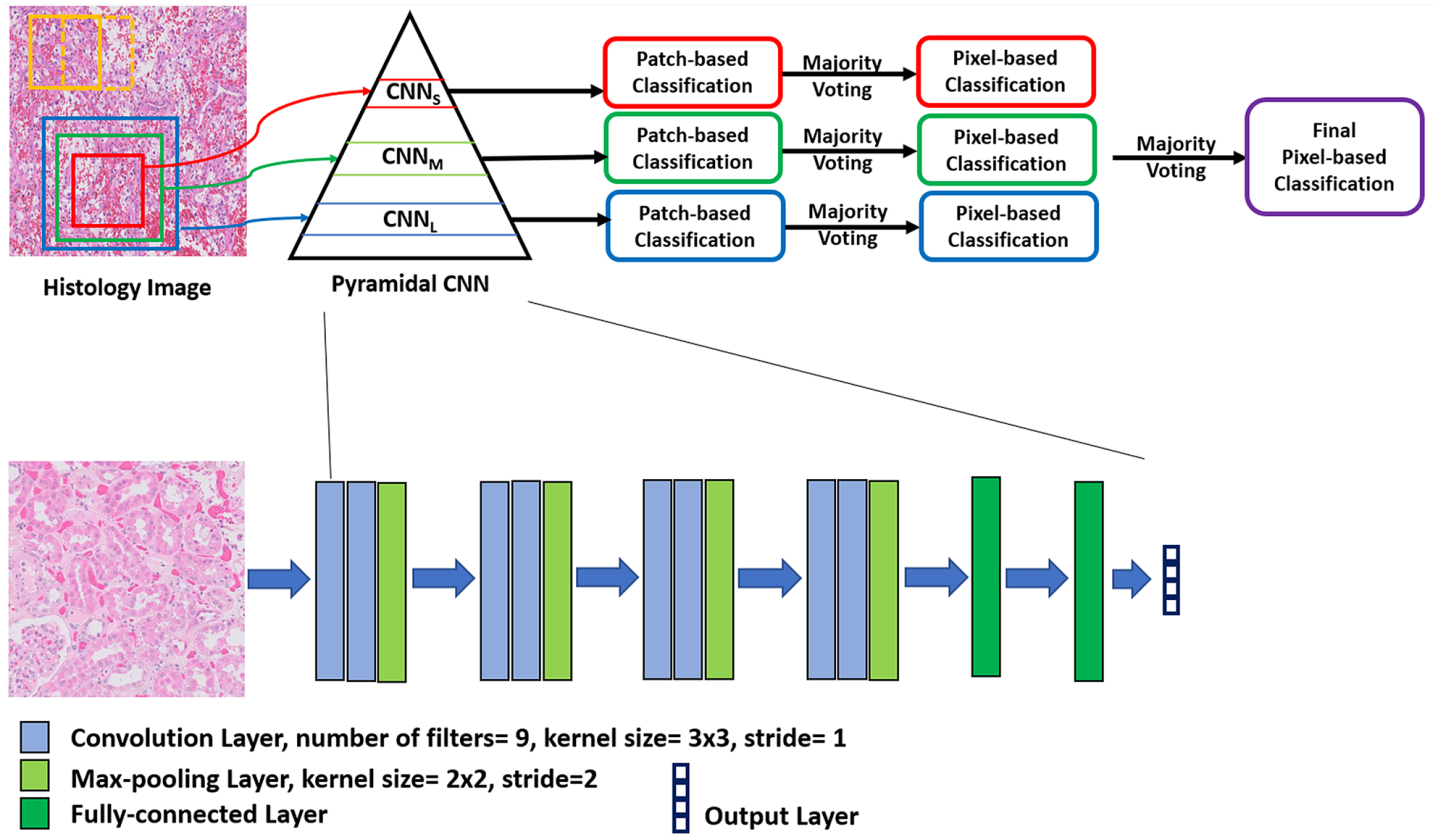
The pyramidal deep learning framework which consists of three convolutional networks for the automated classification of kidney histopathological images.

The proposed deep learning framework (Fig. 4) is composed of three CNNs. Each CNN was designed to process a specific patch size. We referred to the three CNNs by *CNN*_*S*_, *CNN*_*M*_, and *CNN*_*L*_ which processed patches with small (250 × 250), medium (350 × 350), and large (450 × 450) sizes, respectively. We designed the three CNNs to have the same architecture except the input size. The architecture of our CNNs was composed of a series of convolutional blocks where each block contained two convolutional layers followed by a max-pooling layer. After convolutional blocks, there were two fully connected layers. Finally, the output of the fully connected layers was fed to a soft-max layer. The convolutional layer extracted feature maps by convolving the input image with a group of trainable kernels/filters. The feature maps contained features that described the input objects in the input image. Each convolutional layer produced a volume of feature maps because we used a filter group. In our design, filters of size 3 × 3 and stride of 1 were used. The spatial dimensions of the feature maps were reduced by a factor of two in max-pooling layers. The max-pooling layers kept the most prominent features and discarded those less important. Furthermore, max-pooling layers reduced the training time and the computational cost. We used a stride of 2 in max-pooling layers. To summarize, each CNN contains 8 convolutional layers and 4 max-pooling layers. The first fully connected layer was composed of twelve neurons and the second fully connected layer was composed of four neurons for four class classification. The purpose of the soft-max layer is to take the output of the last fully connected layer and convert it to class probabilities in the range of zero to one. The neuron which has the highest probability is the classification result for the input image patch. Table 1 shows our CNN configuration for an input patch of size 250 × 250. Similarly, the same concepts can be applied on the other patch sizes. Each CNN was trained by finding the minimum of the cross-entropy loss. In this loss, the cross-entropy between the predicted class probabilities and the ground truth labels is minimized. The cross-entropy loss is defined as follows:

2 $$ LBCE = - \sum\limits_{i = 1}^{M} {y_{o,i} \;\log (P_{o,i} )} $$

**Table 1:** The proposed CNN configuration for an input patch of size 250 × 250.

Layer	Depth	kernel	Stride	Spatial Size	Parameters
Input	3	–	–	250 × 250 × 3	0
1. Conv	9	3 × 3	1 × 1	248 × 248 × 9	3 × 3 × 3 × 9
2. Conv	9	3 × 3	1 × 1	246 × 246 × 9	3 × 3 × 9 × 9
3. Max-pool	9	2 × 2	2 × 2	123 × 123 × 9	0
4. Conv	9	3 × 3	1 × 1	121 × 121 × 9	3 × 3 × 9 × 9
5. Conv	9	3 × 3	1 × 1	119 × 119 × 9	3 × 3 × 9 × 9
6. Max-pool	9	2 × 2	2 × 2	59 × 59 × 9	0
7. Conv	9	3 × 3	1 × 1	57 × 57 × 9	3 × 3 × 9 × 9
8. Conv	9	3 × 3	1 × 1	55 × 55 × 9	3 × 3 × 9 × 9
9. Max-pool	9	2 × 2	2 × 2	27 × 27 × 9	0
10. Conv	9	3 × 3	1 × 1	25 × 25 × 9	3 × 3 × 9 × 9
11. Conv	9	3 × 3	1 × 1	23 × 23 × 9	3 × 3 × 9 × 9
12. Max-pool	9	2 × 2	2 × 2	11 × 11 × 9	0
13. Concat	1	–	–	1089 × 1	0
14. Full	1	–	–	12 × 1	1089 × 12
15. Full	1	–	–	4 × 1	12 × 4
16. Softmax	1	–	–	4 × 1	0

Total number of parameters = 18,462.

where M is the number of classes. *y*_*o,i*_ is a binary indicator (0 or 1) which indicates the correct classification that observation *o* belongs to class *i*. *P*_*o,i*_ is the predicted probability that observation *o* belongs to class *i*. To overcome network overfitting, a drop out was used with a rate 0.2 in the convolutional and fully connected layers.

#### Output of the deep learning framework

The input image patches are classified to one of the four classes. Then, we assembled the classification of the patches to obtain a classification map for the whole-slide image. Each CNN in our deep learning framework can provide a patchwise and pixelwise accuracy. Our CNNs provide patchwise accuracy because it can classify the input patch to one of the four classes. For one slide image, the patchwise accuracy is defined as follows:

3 $$ {\text{Patchwise}}\;{\text{Accuracy = }}\frac{{{\text{number}}\;{\text{of}}\;{\text{correctly}}\;{\text{classified}}\;{\text{patches}}}}{{{\text{total}}\;{\text{number}}\;{\text{of}}\;{\text{patches}}}} $$

We estimated the patchwise accuracy for all slide images. Then, we estimated the average accuracy along slide images.

Patches of the second dataset had a five-pixels shift between each other. Therefore, each pixel in the image can belong to several patches. Normally, when a single patch was classified, a label was assigned; the same label was given to the pixels of this patch. When we assign the patch label to all pixels in the patch, the background was checked for pixels (pixels with green color) in the patch. Then, the background pixels were assigned a background label which was kept until the final labeling. Given that pixels belong to many patches, each pixel could have several labels. We used majority voting to convert the several labels of a pixel into one label. Then, a labeled slide image (on the pixel level) was created for each slide image. For a single slide image, the pixelwise accuracy was defined as follows:

4 $$ {\text{Pixelwise}}\;{\text{Accuracy}} = \frac{{{\text{number}}\;{\text{of}}\;{\text{ correctly}}\;{\text{classified}}\;{\text{pixels}}}}{{{\text{total}}\;{\text{number}}\;{\text{of}}\;{\text{pixels}}}} $$

The average of accuracies for all slide images was then estimated. To get an improved pixelwise accuracy, we combined the result of the three CNNs. Given that our framework contains three CNNs, we obtained three labels for each pixel in an inputted slide image. Again, we adopted a majority voting strategy to get one label for each pixel. Finally, the final pixelwise accuracy and the average accuracy over all slide images were estimated after combining CNNs results.

#### Generalized Gauss-Markov random field smoothing

We assigned a label for each pixel in the slide image, resulting in a labeled image for the WSI. To preserve continuity and remove inconsistencies (smooth) the labeled image, we considered the estimated labels (denoted *δ*) as samples generated from a generalized 2-D GGMRF model^9^. Each pixel had an eight-neighborhood voxel set (Supplemental Fig. S2). The voxelwise relaxation22 and maximum a posteriori estimates amplified the continuity of the δ values:

5 $$ \begin{gathered} \hat{\delta }_{s} = \arg \min \left\{ {\left| {\delta_{s} - \hat{\delta }_{s} } \right|^{\alpha } + \rho^{\alpha } \lambda^{\beta } \sum {\eta_{s,r} } \left| {\delta_{s} - \hat{\delta }_{s} } \right|^{\beta } } \right\} \hfill \\ \hat{\delta }_{s} \;\;\;r \in vs \hfill \\ \end{gathered} $$

where the original label and its expected estimates are denoted by *δ*_*s*_ and *δ*^ˆ^_*s*_, respectively, at *s* = (*x,y*) which is the observed 2D location. The GGMRF potential is denoted by *η*_*s,r*_. The eight-neighborhood voxel set is denoted by *ν*_*s*_ (Supplemental Fig. S2). *ρ* and *λ* are scaling factors. *α* ∈ 1*,*2 is a parameter that determines the Laplace (*α* = 1) or the gaussian (*α* = 2) estimator’s prior√ distribution. *β* is a parameter that controls the degree of smoothness. We set *α* = 2, *β* = 1*.*01, *ρ* = 1, *λ* = 5, and *η*_*s,r*_ = 2. We hypothesized that GGMRF smoothing could increase the pixelwise accuracy of the labeled slide image.

Algorithm 1 summarizes how our deep learning framework produced a pixelwise classification map for the tested slide image and the estimated classification accuracy. The average accuracy over all slide images is reported in the “Results” section.

## Results

The deep learning library, TensorFlow^10^, was used to develop our deep learning framework. The parameters of our deep learning framework, such as network architecture, should be optimized to obtain the optimal accuracy. We performed this optimization process using grid search strategy by searching for the framework parameters that gave the best system performance. The searched parameters were: (1) the number of convolutional layers, (2) kernel size, (3) initialization of the convolutional kernels, (4) number of filters, (5) stride, (6) patch size, (7) number of epochs, (8) learning rate, and (9) type of optimizer. The best parameters from the grid search are presented in Table 2. During training of our deep learning framework, we kept 20% of the training data for validation. After each epoch, we estimated the validation accuracy and validation loss. The best final model was the one that gave the highest validation accuracy. Data augmentation was a way to increase training data to avoid overfitting. We adopted a data augmentation strategy that consisted of random rotation, scaling, and flipping.

**Table 2:** Optimal parameters of our deep learning framework.

Parameter	Value
Number of convolutional layers	8
Kernel size	3 × 3
Kernel initialization	He initialization^11^
Number of filters	9
Stride	1 (convolution), 2 (max-pooling)
Patch size	32
Number of epochs	60
Learning rate	0.001
Optimizer	Adam

**Figure 5:**
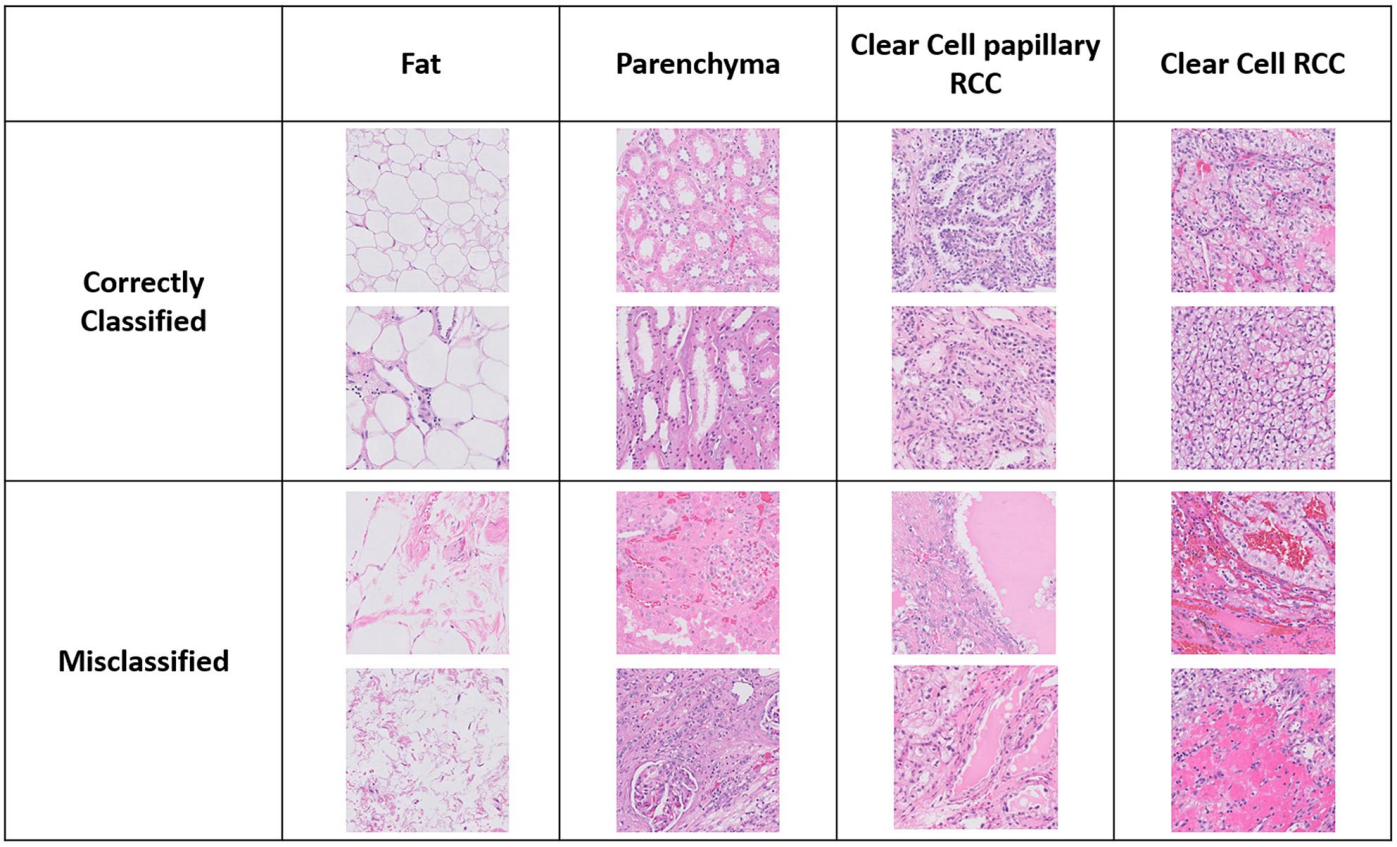
Samples of correctly and wrongly classified patches for the four.

We trained our framework by 70% of the patches of the first dataset and the remaining 30% was used for testing. During training and testing, each CNN of the three CNNs was fed by the appropriate patch size. After testing, we calculated the patchwise accuracy for the four tissue types. Supplemental Table S2 shows the patchwise accuracy for each tissue type. Furthermore, Fig. 5 shows samples of correctly and wrongly classified patches for the four kidney tissue types.
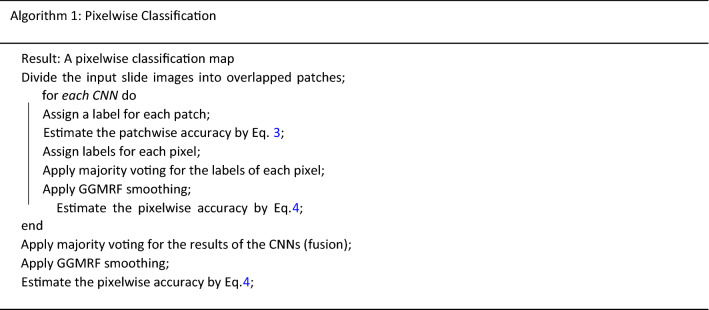

After testing the framework using the testing set, we performed final validation using the second dataset. In the second dataset we made a five-pixels shift between successive patches to perform pixelwise classification in addition to the patchwise classification. The second dataset was fed to our framework one slide at time, dividing the slide image into the three patch sizes, and performed patch and pixel classification for that slide image. The average accuracy over slide images for each tissue type was recorded. Supplemental Table S3 shows the average of patchwise accuracy and the average of pixelwise accuracy of fat, parenchyma, clear cell papillary RCC, and clear cell RCC cases. The classifications of the three CNNs were combined. Each pixel in the tested slide image could have three labels and by applying majority voting we obtain one label for each pixel. Then, we estimated the pixelwise accuracy for the labeled slide image. To remove inconsistencies, we applied GGMRF smoothing on the labeled slide image. To assess the performance of our method after obtaining the final labeling, we constructed a confusion matrix from which we estimated the accuracy, sensitivity, and specificity for detecting each tissue type. Supplemental Table S4 shows the confusion matrix of our proposed method and Supplemental Table S5 shows the performance metrics for detecting each tissue type.

To compare the performance of our proposed approach with other deep learning models, we used two pre-trained models: ResNet18 and ResNet34. In these two models, we replaced the last layers with two output layers. Then, fine-tuning was performed using the kidney data. Supplemental Tables S6, S7 show the confusion matrices for the labeling resulted from the use of ResNet18 and ResNet 34, respectively. Table 3 demonstrates a quantitative comparison in terms of accuracy, sensitivity, and specificity between our framework and ResNets. We performed a *t*-test to show that there is a significant difference between our method of approach and ResNets’. We obtained a *P*-value of 0.01 for ResNet18 and a *P*-value of 0.02 for ResNet34. The *P*-values are less than 0.05. Therefore, the null hypothesis can be rejected in the two tests. We can conclude that the differences between our method and other models are statistically significant. Supplemental Figures S3, Fig. S4, Fig. S5, and Fig. S6 show the labeling on the pixel level for the four tissue types.

**Table 3:** Quantitative comparison between our approach and other deep learning models. The values are the estimated averages across tissue types.

	Average accuracy	Average sensitivity	Average specificity
ResNet18	0.942	0.892	0.961
ResNet34	0.937	0.882	0.957
Proposed without GGMRF smoothing	0.946	0.911	0.965
Proposed with GGMRF smoothing	0.957	0.920	0.971

## Discussion

A computer-aided diagnostic system that can automatically classify kidney histopathological images was proposed in this paper. We targeted the challenging task of classifying clear cell papillary RCC and clear cell RCC. The morphological features of these tumor subtypes overlap; however, they have different prognoses. Therefore, our task is necessary to best determine the appropriate clinical management. We used deep learning algorithms to build our system of classification. Our design of pyramidal CNN successfully determined the normal tissues and abnormal tissues and managed to differentiate between clear cell papillary RCC and clear cell RCC. Our approach also generated classification maps that classify the kidney histopathological images on the pixel-level. Finally, our method removed inconsistencies from the generated maps using GGMRF smoothing.

An enormous amount of information can be found in the whole slide histopathological images. The pathologist spends significant time and effort to manually examine the histology image. As there is a growth in the number of diagnosed cancer cases, fast and accurate analysis of histology images is needed. Our study addresses this shortcoming by proposing a computer-aided diagnostic system that can automatically classify kidney tissues and detect kidney cancers. Our approach can be adopted to the diagnosis of other cancers.

As shown in Supplemental Table S3, our approach gave a high patchwise accuracy. In other words, our framework can determine tissue types from image patches with different sizes. Patch size of 350 × 350 resulted in the best patchwise accuracy. For the cases of fat, parenchyma, and clear cell papillary RCC, patch size of 350 × 350 resulted in better patchwise accuracy. For the case of clear cell RCC, patch sizes of 350 × 350 and 450 × 450 resulted in similar patchwise accuracy; however, patch size of 350 × 350 gave better accuracy than patch size of 250 × 250.

Our pyramidal framework allowed us to analyze the histopathological images at various spatial scales. Then, we combined the classifications of the three CNNs to obtain a better pixelwise classification. Combining the results allowed us to get better accuracy than from a single CNN. Furthermore, we can provide the pathologist the opportunity to analyze the pathology regions at small scale because our framework resulted in pixel-level classification. Patch size of 350 × 350 gave the best pixelwise accuracy. For the cases of parenchyma and clear cell RCC, better pixelwise accuracy could be obtained from patch size of 350 × 350. Similar pixelwise accuracy resulted from patches with sizes 350 × 350 and 450 × 450 for the cases of fat and clear cell papillary RCC; however, patch size of 350 × 350 had better accuracy than patch size of 250 × 250.

Supplemental Table S5 shows that the fusion of CNN classifications produced good classification for the pixels in terms of accuracy, sensitivity, and specificity. After fusion, we obtained the best performance metrics from fat cases while clear cell papillary RCC and clear cell RCC gave the lowest performance metrics. We can conclude that CNNs extract new features related to the underlying tissue texture from each patch size and fusing between CNN classifications resulted in enhancing the final classification.

Finally, using GGMRF smoothing enhanced the labeling of the input slide image as it worked on removing inconsistencies, (Table 3). Furthermore, our deep learning framework surpassed other state-of-the-art models, such as ResNet18 and ResNet34, demonstrating that the notion of fusing CNN decisions is fruitful for obtaining improved classification.

## Supplementary Information

Supplementary Legends.

Supplementary Tables.

Supplementary Figure 1.

Supplementary Figure 2.

Supplementary Figure 3.

Supplementary Figure 4.

Supplementary Figure 5.

Supplementary Figure 6.

## Data Availability

The datasets generated during and/or analyzed during the current study are available from the corresponding author on a reasonable request.
