# Author Correction: A pyramidal deep learning pipeline for kidney whole-slide histology images classification

**DOI:** 10.1038/s41598-021-01416-x

**Published:** 2021-11-02

**Authors:** Hisham Abdeltawab, Fahmi Khalifa, Mohammed Ghazal, Liang Cheng, Dibson Gondim, Ayman El-Baz

**Affiliations:** 1 Bioengineering Department, University of Louisville, Louisville, KY USA; 2 Department of Pathology, Indiana University School of Medicine, Indianapolis, IN USA; 3 Department of Urology, Indiana University School of Medicine, Indianapolis, IN USA; 4 Department of Pathology and Laboratory Medicine, University of Louisville, Louisville, KY USA; 5 Department of Electrical and Computer Engineering, Abu Dhabi University, Abu Dhabi, United Arab Emirates

Correction to: *Scientific Reports* https://doi.org/10.1038/s41598-021-99735-6, published online 12 October 2021

The original version of this Article contained an error in the spelling of the author Mohammed Ghazal which was incorrectly given as Mohammed Mohammed.

In addition, the author Mohammed Ghazal was incorrectly affiliated with ‘Bioengineering Department, University of Louisville, Louisville, KY, USA”, “Department of Pathology, Indiana University School of Medicine, Indianapolis, IN, USA” and “Department of Urology, Indiana University School of Medicine, Indianapolis, IN, USA.” The correct affiliation is listed below.

Department of Electrical and Computer Engineering, Abu Dhabi University, Abu Dhabi, United Arab Emirates.

The original Article has been corrected.

