# Supplementary material for: A pyramidal deep learning pipeline for kidney whole-slide histology images classification
Source: Sci Rep. 2021 Oct 12;11:20189. doi: 10.1038/s41598-021-99735-6 (PMC8511039; doi:10.1038/s41598-021-99735-6)
Supplement: Supplementary file 1 — Supplementary Legends. [file 41598_2021_99735_MOESM1_ESM.docx]

**Supplemental Figures Legends**

**Supplementary Figure S1.** Weights of employed sharpening filter.

**Supplementary Figure S2.** For eight neighbors, the figure shows the pairwise voxel interaction in a 2-D GGMRF image model.

**Supplementary Figure S3.** (I) Whole slide image with fat. (II) is zoomed region, (III) is the labeled region before GGMRF smoothing, and (IV) is the labeled region after GGMRF smoothing. Green (Class 1), yellow (Class 2), red (Class 3), and blue (Class 4) refer to fat, parenchyma, clear cell papillary RCC, and clear cell RCC, respectively.

**Supplementary Figure S4.** (I) Whole slide image with kidney parenchyma. (II) is zoomed region, (III) is the labeled region before GGMRF smoothing, and (IV) is the labeled region after GGMRF smoothing. Green (Class 1), yellow (Class 2), red (Class 3), and blue (Class 4) refer to fat, parenchyma, clear cell papillary RCC, and clear cell RCC, respectively.

**Supplementary Figure S5.** (I) Whole slide image with clear cell papillary RCC. (II) is zoomed region, (III) is the labeled region before GGMRF smoothing, and (IV) is the labeled region after GGMRF smoothing. Green (Class 1), yellow (Class 2), red (Class 3), and blue (Class 4) refer to fat, parenchyma, clear cell papillary RCC, and clear cell RCC, respectively.

**Supplementary Figure S6.** (I) Whole slide image with clear cell RCC. (II) is zoomed region, (III) is the labeled region before GGMRF smoothing, and (IV) is the labeled region after GGMRF smoothing. Green (Class 1), yellow (Class 2), red (Class 3), and blue (Class 4) refer to fat, parenchyma, clear cell papillary RCC, and clear cell RCC, respectively.
