# Supplementary material for: A pyramidal deep learning pipeline for kidney whole-slide histology images classification
Source: Sci Rep. 2021 Oct 12;11:20189. doi: 10.1038/s41598-021-99735-6 (PMC8511039; doi:10.1038/s41598-021-99735-6)
Supplement: Supplementary file 2 — Supplementary Tables. [file 41598_2021_99735_MOESM2_ESM.docx]

**Supplementary Tables**

**Supplementary Table S1**

| WSI tissue classification | Set 1 | | Set 2 |
| --- | --- | --- | --- |
|  | # Training slides | # Testing slides | # Validation slides |
| Class 1: Fat | 2 | 1 | 2 |
| Class 2: Parenchyma | 4 | 1 | 2 |
| Class 3: Clear cell papillary RCC | 14 | 2 | 6 |
| Class 4: Clear cell RCC | 18 | 2 | 10 |

**Supplementary Table S2.** The estimated patchwise accuracy for the testing set. The table shows the accuracy for the four tissue types at different patch sizes.

| Tissue Type | Patch Size | | |
| --- | --- | --- | --- |
|  | 250×250 | 350×350 | 450×450 |
| Fat | 0.89 ± 0.01 | 0.91 ± 0.02 | 0.90 ± 0.11 |
| Parenchyma | 0.88 ± 0.11 | 0.91 ± 0.12 | 0.90 ± 0.24 |
| Clear cell papillary RCC | 0.86 ± 0.23 | 0.90 ± 0.21 | 0.89 ± 0.21 |
| Clear cell RCC | 0.85 ± 0.02 | 0.89 ± 0.31 | 0.89 ± 0.03 |

**Supplementary Table S3.** For the fat, parenchyma, clear cell papillary RCC, and clear cell RCC cases of the second dataset, we estimated the average patchwise accuracy and the pixelwise accuracy. The table shows the accuracy at different patch sizes.

| Tissue |  | Patch Size | | |
| --- | --- | --- | --- | --- |
|  |  | 250×250 | 350×350 | 450×450 |
| Fat | patchwise | 0.87 ± 0.04 | 0.90 ± 0.11 | 0.89 ± 0.05 |
|  | pixelwise | 0.89 ± 0.01 | 0.91 ± 0.08 | 0.91 ± 0.11 |
| Parenchyma | patchwise | 0.85 ± 0.11 | 0.88 ± 0.21 | 0.87 ± 0.03 |
|  | pixelwise | 0.88 ± 0.05 | 0.90 ± 0.08 | 0.89 ± 0.16 |
| Clear cell papillary RCC | patchwise | 0.84 ± 0.14 | 0.87 ± 0.06 | 0.86 ± 0.07 |
|  | pixelwise | 0.86 ± 0.11 | 0.90 ± 0.05 | 0.90 ± 0.21 |
| Clear cell RCC | patchwise | 0.83 ± 0.20 | 0.86 ± 0.01 | 0.86 ± 0.02 |
|  | pixelwise | 0.85 ± 0.05 | 0.89 ± 0.04 | 0.88 ± 0.11 |

**Supplementary Table S4.** Confusion matrix based on the final labeling obtained from our proposed approach, where Class 1, Class 2, Class 3, and Class 4 refer to fat, parenchyma, clear cell papillary RCC, and clear cell RCC, respectively. Values are shown as percentages.

| Actual label | Predicted Label | | | | |
| --- | --- | --- | --- | --- | --- |
|  |  | Class 1 | Class 2 | Class 3 | Class 4 |
|  | Class 1 | 92.98% | 2.68% | 1.75% | 1.69% |
|  | Class 2 | 2.22% | 92.41% | 3.75% | 2.66% |
|  | Class 3 | 1.92% | 2.51% | 91.35% | 4.38% |
|  | Class 4 | 2.87% | 2.41% | 3.15% | 91.30% |

**Supplementary Table S5.** Performance metrics for the labeling obtained from our framework for each tissue type. The values in the table are based on the confusion matrix in Supplementary Table S4.

| Tissue | Accuracy | Sensitivity | Specificity |
| --- | --- | --- | --- |
| Fat | 0.98 | 0.93 | 0.98 |
| Parenchyma | 0.97 | 0.92 | 0.97 |
| Clear cell papillary RCC | 0.95 | 0.91 | 0.96 |
| Clear cell RCC | 0.94 | 0.91 | 0.97 |

**Supplementary Table S6.** Confusion matrix based on the labeling obtained from ResNet18, where Class 1, Class 2, Class 3, and Class 4 refer to fat, parenchyma, clear cell papillary RCC, and clear cell RCC, respectively. Values are shown as percentages.

| Actual label | Predicted Label | | | | |
| --- | --- | --- | --- | --- | --- |
|  |  | Class 1 | Class 2 | Class 3 | Class 4 |
|  | Class 1 | 90.13% | 3.78% | 3.31% | 3.08% |
|  | Class 2 | 3.52% | 90.00% | 4.70% | 4.04% |
|  | Class 3 | 2.59% | 3.13% | 88.50% | 4.87% |
|  | Class 4 | 3.75% | 3.09% | 3.49% | 88.00% |

**Supplementary Table S7.** Confusion matrix based on the labeling obtained from ResNet34, where Class 1, Class 2, Class 3, and Class 4 refer to fat, parenchyma, clear cell papillary RCC, and clear cell RCC, respectively. Values are shown as percentages.

| Actual label |  | Predicted Label | | | |
| --- | --- | --- | --- | --- | --- |
|  |  | Class 1 | Class 2 | Class 3 | Class 4 |
|  | Fat | 89.10% | 4.31% | 2.91% | 2.78% |
|  | Parenchyma | 3.83% | 89.00% | 5.94% | 2.87% |
|  | Clear cell papillary RCC | 2.99% | 3.79% | 87.50% | 7.35% |
|  | Clear cell RCC | 4.08% | 2.89% | 3.66% | 87.00% |
