# Supplementary figures and images for: A pyramidal deep learning pipeline for kidney whole-slide histology images classification
Source: Sci Rep. 2021 Oct 12;11:20189. doi: 10.1038/s41598-021-99735-6 (PMC8511039; doi:10.1038/s41598-021-99735-6)

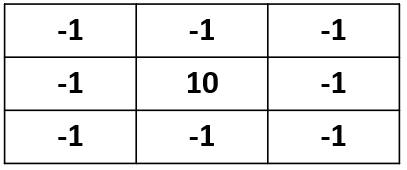

Supplement: Supplementary file 3 — Supplementary Figure 1. [file 41598_2021_99735_MOESM3_ESM.png]

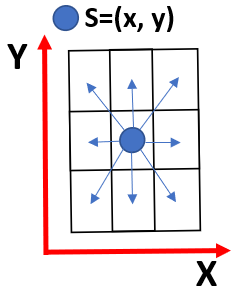

Supplement: Supplementary file 4 — Supplementary Figure 2. [file 41598_2021_99735_MOESM4_ESM.png]

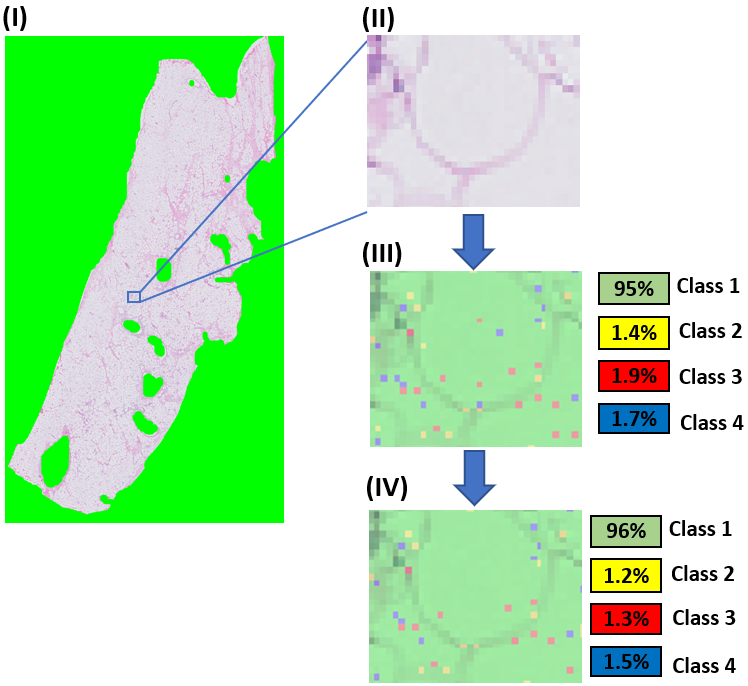

Supplement: Supplementary file 5 — Supplementary Figure 3. [file 41598_2021_99735_MOESM5_ESM.png]

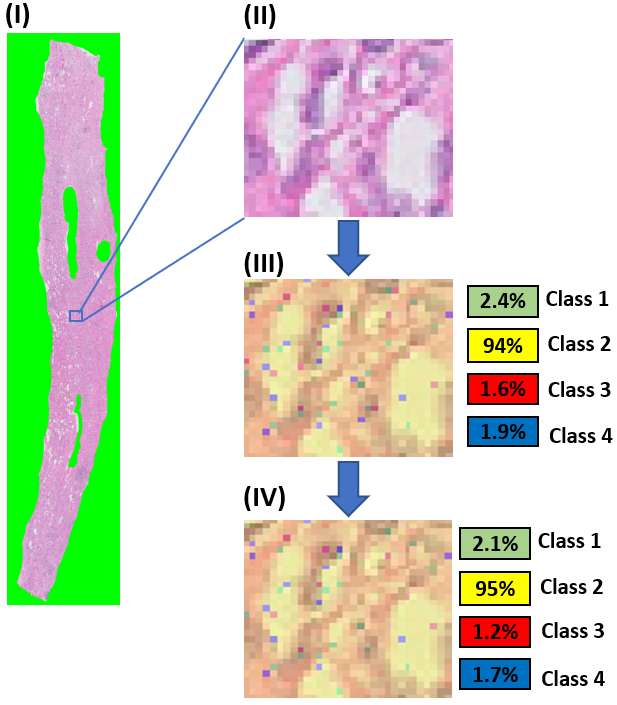

Supplement: Supplementary file 6 — Supplementary Figure 4. [file 41598_2021_99735_MOESM6_ESM.png]

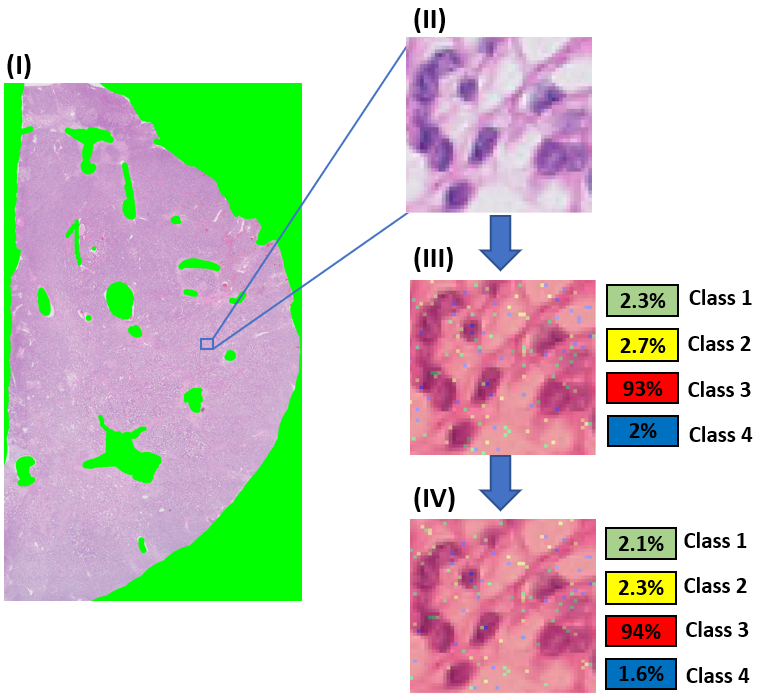

Supplement: Supplementary file 7 — Supplementary Figure 5. [file 41598_2021_99735_MOESM7_ESM.png]

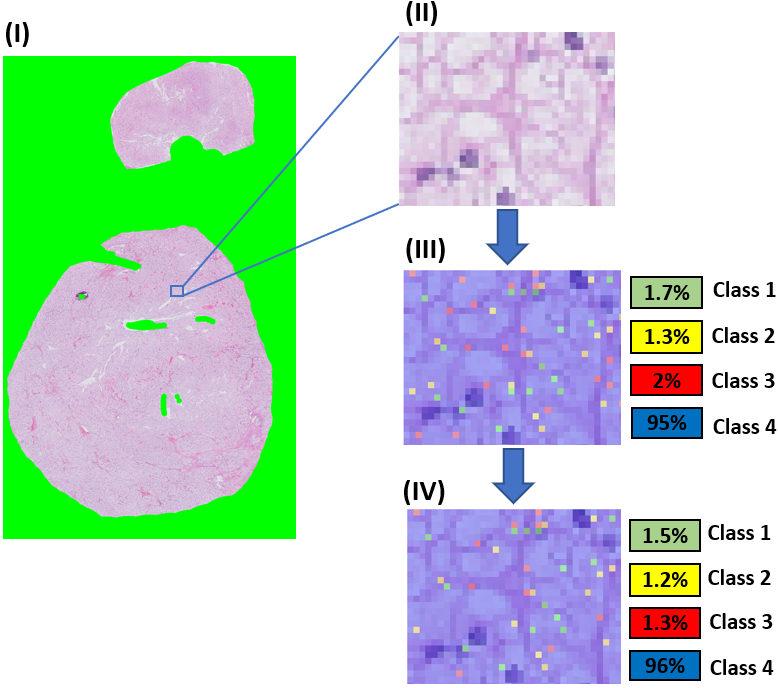

Supplement: Supplementary file 8 — Supplementary Figure 6. [file 41598_2021_99735_MOESM8_ESM.png]
